# A high-adaptability nozzle-array printing system based on a set covering printing planning model for printed display manufacturing

**DOI:** 10.1038/s41598-022-24135-3

**Published:** 2023-01-04

**Authors:** Yixin Wang, Jiankui Chen, Zhouping Yin, Yiqun Li

**Affiliations:** State Key Laboratory of Digital Manufacturing Equipment and Technology, School of Mechanical Science and Engineering, Huazhong University of Science and Technology, Wuhan, 430074 People’s Republic of China

**Keywords:** Mechanical engineering, Applied mathematics, Design, synthesis and processing

## Abstract

Inkjet printing technology is expected to enhance printed display mass production technology in the future. Nozzle-array printheads form the basis for printed display mass production applications. However, jet instability caused by air bubble entrapment and nozzle wettability changes during the printing process is a major challenge in the application of this technology. To adapt to possible nozzle abnormalities, a high-adaptability nozzle-array printing system based on a set covering printing planning (SCPP) model for printed display manufacturing is designed in this study. The study consists of two parts. First, a printing system based on multistep visual inspection and closed-loop feedback is proposed to accurately detect and screen abnormal nozzle positions. Notably, the inkjet printing system can identify nozzles with abnormal ejection characteristics and ensure that the remaining nozzles work accurately and stably. Then, an SCPP model is established for display pixel printing planning by using the remaining normal nozzles on the nozzle-array printhead. This model can output the most efficient printing path and nozzle printing action and can adapt to any pixel pattern, nozzle type, and abnormal nozzle distribution. The system and technology are highly adaptable and scalable for fabricating large-area printed display devices.

## Introduction

**Figure 1:**
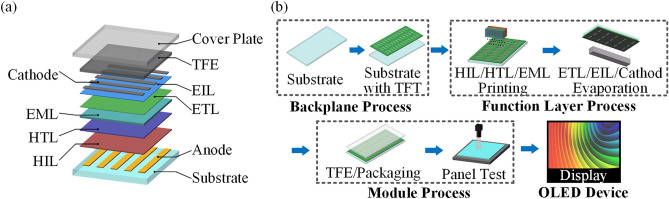
Simplified diagram of the OLED structure and production flow.

Inkjet printing is a technology that can directly deposit a material solution on a substrate to form patterns at room temperature. This approach is considered a next-generation manufacturing method for printed display fabrication due to its low cost, high manufacturing efficiency, and advantages in manufacturing large-area flexible panels^1–3^. Figure 1a shows the typical structure^4^ of an organic light-emitting diode (OLED) device that can be printed. The flow of the printing manufacturing process for this printed display is shown in Fig. 1b. The feasibility of inkjet printing technology in the production of printed displays has been verified by applying such technology in the laboratory. For example, the hole injection layer^5^ (HIL), hole transport layer^6^ (HTL), emission layer^7^ (EML), and thin film encapsulation^8^ (TFE) layer of OLEDs have been prepared by printing.

**Figure 2:**
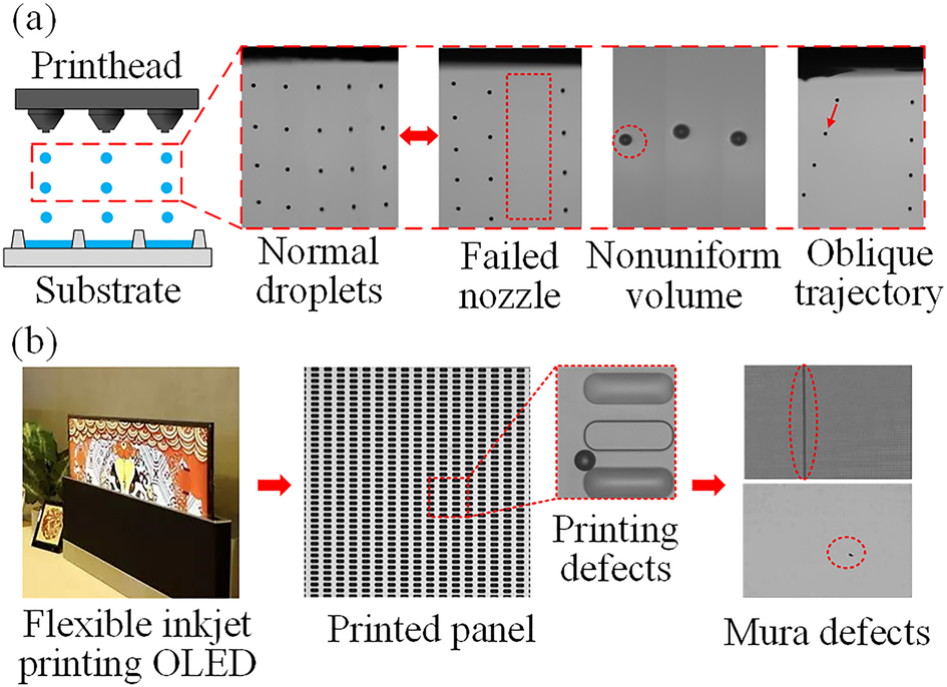
The challenge of using nozzle arrays in display pixel printing. (**a**) Inkjet printing and typical problems. (**b**) Flexible inkjet printing OLED screen released by TCL at the Consumer Electronics Show in 2020 and example defects in a printed panel.

One of the key technologies for printed display mass production is the use of nozzle-array printheads, which are usually composed of one or more rows of multiple nozzles. The nozzles in the same row are arranged at equal intervals, and the nozzles in different rows are arranged in a staggered pattern to improve the printing density. Most production printer systems use nozzle-array printheads, which have thousands of nozzles, to improve the efficiency of inkjet printing^9^. However, for inkjet printing to transition from research and development (R&D) to mass production, one of the greatest challenges in nozzle-array printhead applications is the instability of nozzle ejection, as shown in Fig. 2a. It is difficult to ensure that all the nozzles of a printhead can provide stable jets during the whole printing process. Ejection problems, such as failed nozzles, nonuniform volumes, and oblique trajectories, may cause printing defects on printed panels and then lead to mura defects^10^, which ultimately affect the quality of the printed display, as shown in Fig. 2b. Ejection problems can result from numerous causes, such as poor jet directionality from nozzle plate wetting, partial fluid clogs inside or outside of a nozzle, and air bubble entrapment in the printhead system^11^. The sources of these problems are very complex and difficult to directly identify. Thus, printed display nozzle-array printing systems must be highly adaptable to possible abnormal nozzle ejection conditions.

To solve this problem, the traditional printing system mainly includes the following three steps. (1) Droplet inspection and identification of abnormal nozzles; (2) printhead adjustment until all nozzles are adjusted to the normal ejection state; and (3) rotating the nozzle-array printhead to match the nozzle pitch and the pixel pitch and using the printing planning algorithm to obtain the printing path and nozzle ejection action^12^. For droplet inspection and printhead adjustment, conventional printing systems reduce the appearance of abnormal nozzles through nozzle hydrophobic treatment, ink formulation optimization, and waveform adjustment of the printhead. Han et al.^13^ used a selective hydrophobic coating of the inkjet head plate to enhance the uniformity of printing. Huang et al.^14^ analyzed the effects of metallic nanoparticle sizes in the ink formulation through droplet impact behaviors and determined that this approach could be used to optimize ink formulation. Jiang et al.^15^ proposed a computational fluid dynamics model to investigate the mechanism of the printing process; the model could guide the quick determination of operation parameters for the desired printing resolution when given a new ink. Cao et al.^16^ controlled the droplet volume deviation within 5% through waveform adjustment according to the droplet observation results from a nozzle-array inkjet printing system and used this printing system to print an area of 40 × 40 mm OLEDs with high uniformity. Yun et al.^17^ added a waveform optimization algorithm to a nozzle-array printing system and controlled the droplet velocity deviation within 6% through feedback adjustment based on measurements obtained with a camera. Then, the inkjet printing system used the adjusted normal nozzle information to plan the movement and jetting action of the printing process through a printing planning algorithm and finally achieved OLED printing. The commonly used printing planning methods include raster printing planning algorithms and vector printing planning algorithms. Lin et al.^18^ proposed a novel printing algorithm based on printhead rotation and an interlaced printing method for OLED display fabrication. Chang et al.^19^ developed an interlace rotation algorithm that can plan the printing path and nozzle ejection action by rotating the printhead angle, and the results showed that the printing resolution can be continuously adjusted from 100 dots per inch (DPI) to 5080 DPI. Phung et al.^20^ designed an encoder processing unit and a vector printing algorithm to plan X–Y printing motions and nozzle ejection actions by producing droplets at an equally spaced distance to prevent a nonuniform linewidth near the endpoints where line printing starts and ends. Kim et al.^21^ presented an algorithm to redefine the printed image resolution according to the pixel pitch of OLEDs and then planned the printing process according to a bitmap pattern.

However, the above printing systems highly depend on the accuracy and stability of all the nozzle ejection states. These printing systems cannot adapt to printing conditions with abnormal nozzles. If any nozzle undergoes abnormal ejection, it may cause printing defects for the printed display panels. Moreover, mura-free printed display printing results require that the total thickness for all pixels is uniform; for example, the thickness variation of each OLED layer must be ≤  ± 0.6% of the mean thickness^22^; therefore, a droplet volume deviation within 5% for all nozzles is insufficient. It is difficult to adjust all nozzles to the normal state through waveform adjustment and ink formulation optimization^22^. Essentially, an R&D-type operating condition is required for all nozzles to provide normal and stable jets, and this condition is not suitable for high-throughput mass production. Therefore, a display pixel printing system that can be adapted to any jetting conditions is worth studying.

Inspired by previous nozzle-array inkjet printing systems, this paper presents a display pixel nozzle-array inkjet printing system based on a set covering printing planning (SCPP) model, which can stably and accurately print any type of pixel pattern when abnormal printhead nozzles are identified and closed. The proposed system consists of two parts. First, a nozzle-array inkjet printing system based on multistep visual inspection is designed to realize full-cycle monitoring and feedback control from droplet ejection to deposition for all nozzles on the printhead and screen abnormal nozzles based on the detection results. Second, an SCPP model for printed display fabrication is proposed to plan the printing path and nozzle ejection actions according to the remaining normal nozzle position and pixel pattern. The issue of nozzle ejection instability is resolved with the two components of the proposed system. The highly adaptable nozzle-array inkjet printing system proposed in this study can satisfy the requirements of commercialization and large-scale printed display fabrication.

## Display pixel nozzle-array inkjet printing system based on a set covering printing planning model

### Printing system design and layout

**Figure 3:**
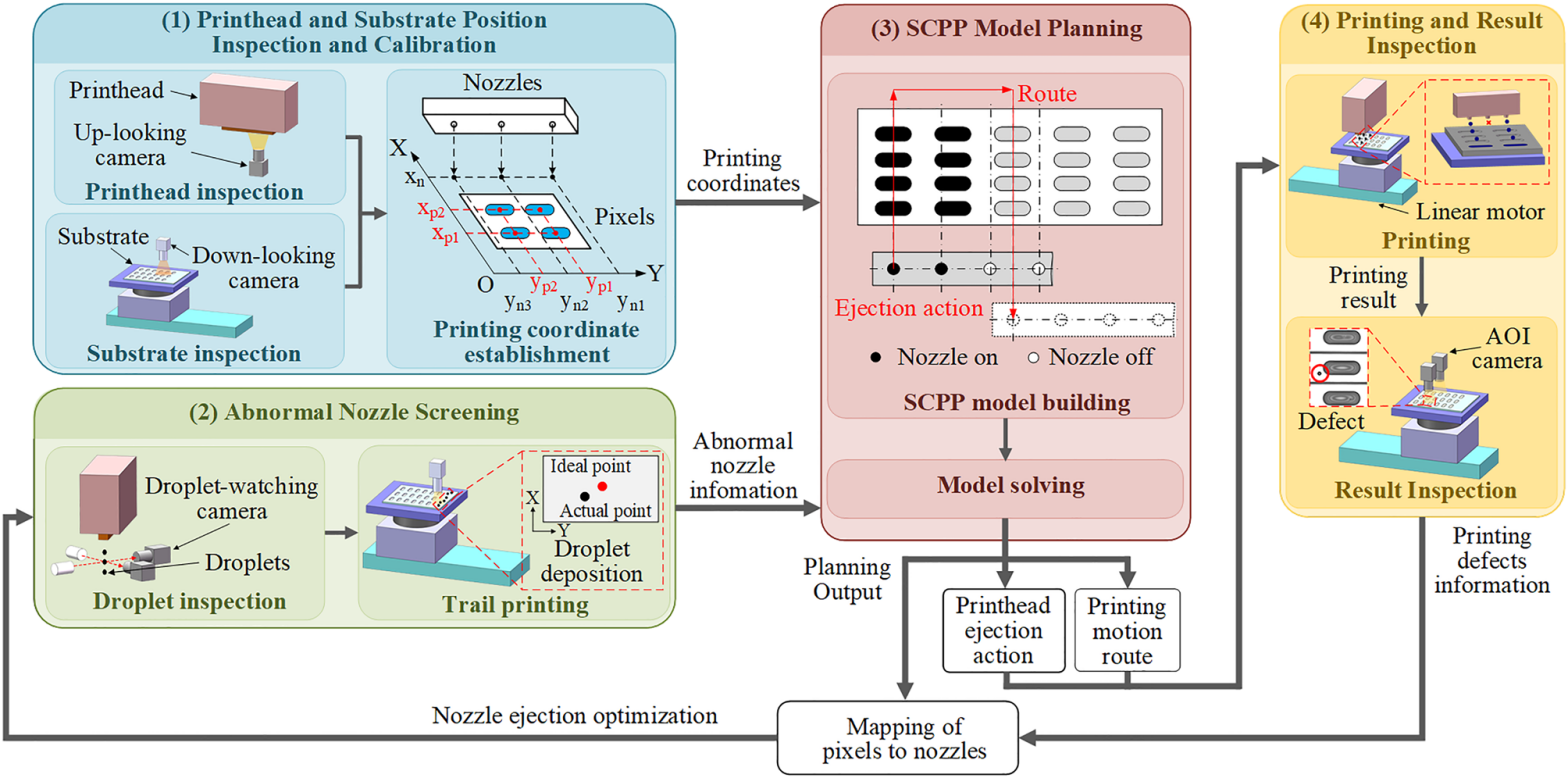
Schematic diagram of the principles of the display pixel inkjet printing system.

The display pixel printing process spans from ejecting droplets from the nozzles of the printhead to deposition in pixels on a substrate. The main objects involved in this process are printheads, droplets, and substrates. The basis of the accurate and stable printing plan includes two points: (1) to determine the coordinates of the nozzle and print pattern and (2) accurately identify the position of abnormal nozzles. Therefore, multistep detection and feedback adjustment must be accurately performed for the printhead, droplets, and substrates. The printing system contains functions such as printhead and substrate inspection, abnormal nozzle screening, printing planning, panel printing, and printing result inspection. The proposed printed display nozzle-array inkjet printing system is divided into four parts according to these functions, as shown in Fig. 3.

1. *Printhead and substrate position inspection and calibration* The printhead installation position is determined with an up-looking camera, and the substrate position is determined with a down-looking camera. If the installation position error exceeds the relevant limit, the printhead and substrate positions are calibrated. After calibration, the coordinate sets for nozzles and the pixels are established in the same coordinate system as used by the printing system.
2. *Abnormal nozzle screening* First, a droplet-watching camera is used to measure the volume, velocity, and angle of droplets ejected from each nozzle. Then, the printhead is used to perform trial printing on the blank position of the substrate, and the down-looking camera is used to measure the error between the actual deposition positions of droplets and the designed positions. According to the measurement results, the printhead driving waveform is adjusted, and printing position compensation is performed to enhance the nozzle ejection results. The serial numbers of abnormal nozzles with droplet parameters that exceed the relevant limits are recorded.
3. *Model planning* According to the recorded abnormal nozzle situation and the printed display pattern requirements, a proposed SCPP model is used to plan the printing process. The output of the model includes the printing motion route, the printhead ejection action, and the mapping between nozzles and pixels. The first two outputs are used to perform substrate printing. The third output is used to find the abnormal nozzle position that causes defects when the defect is found after printing.
4. *Panel printing and result inspection* According to the planned printing route and nozzle ejection actions, a nozzle-array printhead is used to complete the printing of all pixels on the substrate. After the printing is completed, the printing results are inspected with an automated optical inspection (AOI) camera. According to the positions of the inspected printing defects and the mapping between nozzles and pixels, abnormal nozzles are rescreened and closed in the next printing.

**Figure 4:**
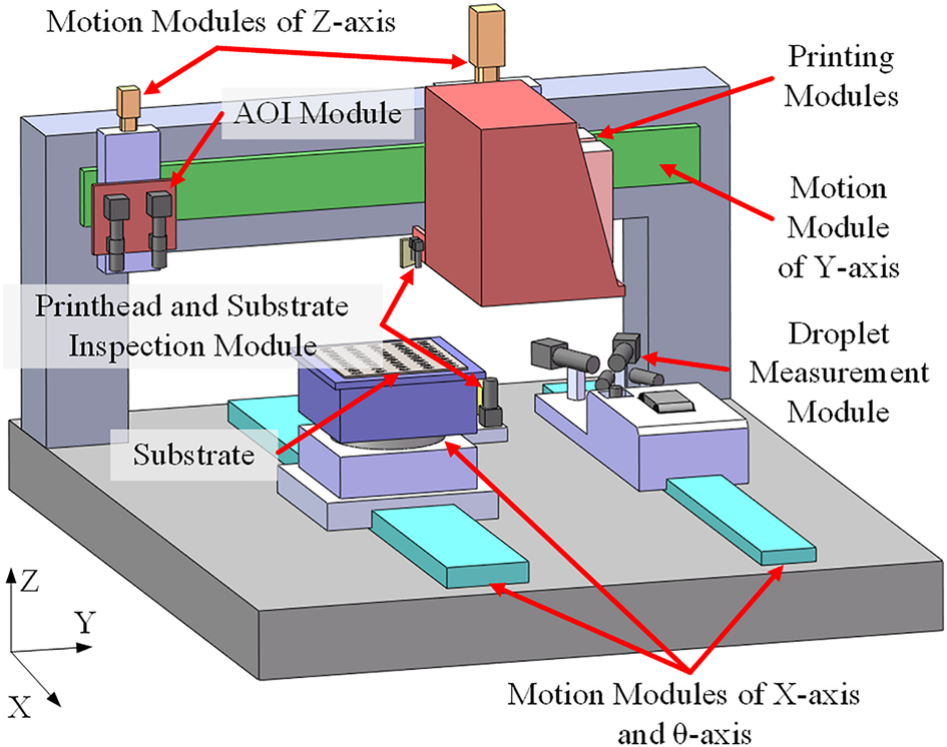
Simplified diagram of the printing system.

Based on the design for the display pixel printing process in Fig. 3, the system needs to include the following modules: (1) a printing module for the stable ejection of droplets; (2) a printhead and substrate inspection module for obtaining position information for all nozzles and pixels; (3) a droplet measurement module for the accurate detection of parameters for droplets ejected by each nozzle, including the droplet volume, speed, angle, and whether there are satellite droplets; (4) an AOI module to assess the printing results and collect printing defect information; and (5) a motion module for the execution of printing actions and the movement of various modules. A prototype of the display pixel nozzle-array printing system is shown in Fig. 4, and it includes the above function modules and a possible equipment layout. The layout advantages include the following: (1) modules are arranged from right to left according to the module transfer sequence during the printing process to improve the conversion efficiency of different modules in this process; (2) the droplet measurement module that requires droplet ejection is arranged separately from the printing area to ensure that ejected droplets do not pollute the substrate; and (3) the use of different motion axes is maximized to save system space.

The printing module includes the printhead, the printing control system, and the ink supply system. The ink supply system guides the ink through the hose and pump to achieve a stable flow from the ink cartridge to the nozzles and ensures that the ink pressure is within a small steady-state error range. The printing control system receives the printhead ejection signal sent from the host computer to achieve ink jetting. The printhead and substrate inspection modules include an up-looking camera and a down-looking camera, respectively. Through these two cameras, the positions of the printhead and the substrate can be detected. According to the position coordinates of the two cameras in the printing system, the position coordinates of all nozzles on the printhead and all pixels on the substrate are established in the coordinate system of the printing system. The droplet measurement module uses a self-developed accurate stereovision-based droplet measurement system^23^; it includes two droplet-watching cameras at different angles on the same horizontal plane, synchronized triggering stroboscopic lights, and multiple image processing algorithms to obtain high-quality three-dimensional images of high-speed flying droplets and accurately measure their volume, speed, and angle. The AOI module contains multiple sets of industrial cameras and coaxial light sources and an automatic recognition algorithm for printing defects. This module can quickly collect images of the printing results and obtain the type and location information for printing defects through image processing. The motion module is composed of a multiaxis motion platform, in which the Y-axis motion platform moves the printhead modules, the substrate inspection module, and the AOI module to the working position; the X-axis motion platform transports the droplet measurement module, printhead inspection module, and adsorption platform on which the substrate is placed; the Z-axis motion platform lifts the printhead module and the AOI module; and the θ-axis motion platform rotates the substrate adsorption platform.

### Closed loops in the printing process flow

Based on the thickness and uniformity requirements of the printed display functional layer and the manufacturing efficiency of the printing system, the performance indexes of this printing system are as follows: (1) printing speed ≥ 100 mm/s; (2) droplet deposition position error ≤  ± 10 μm; and (3) mura-free printing result. Therefore, each step in the proposed process requires closed-loop detection and feedback adjustment to improve the accuracy and stability of the printing system and to provide accurate screening results for abnormal nozzle positions on the printhead for the next step of planning and printing in the process flow.

**Figure 5:**
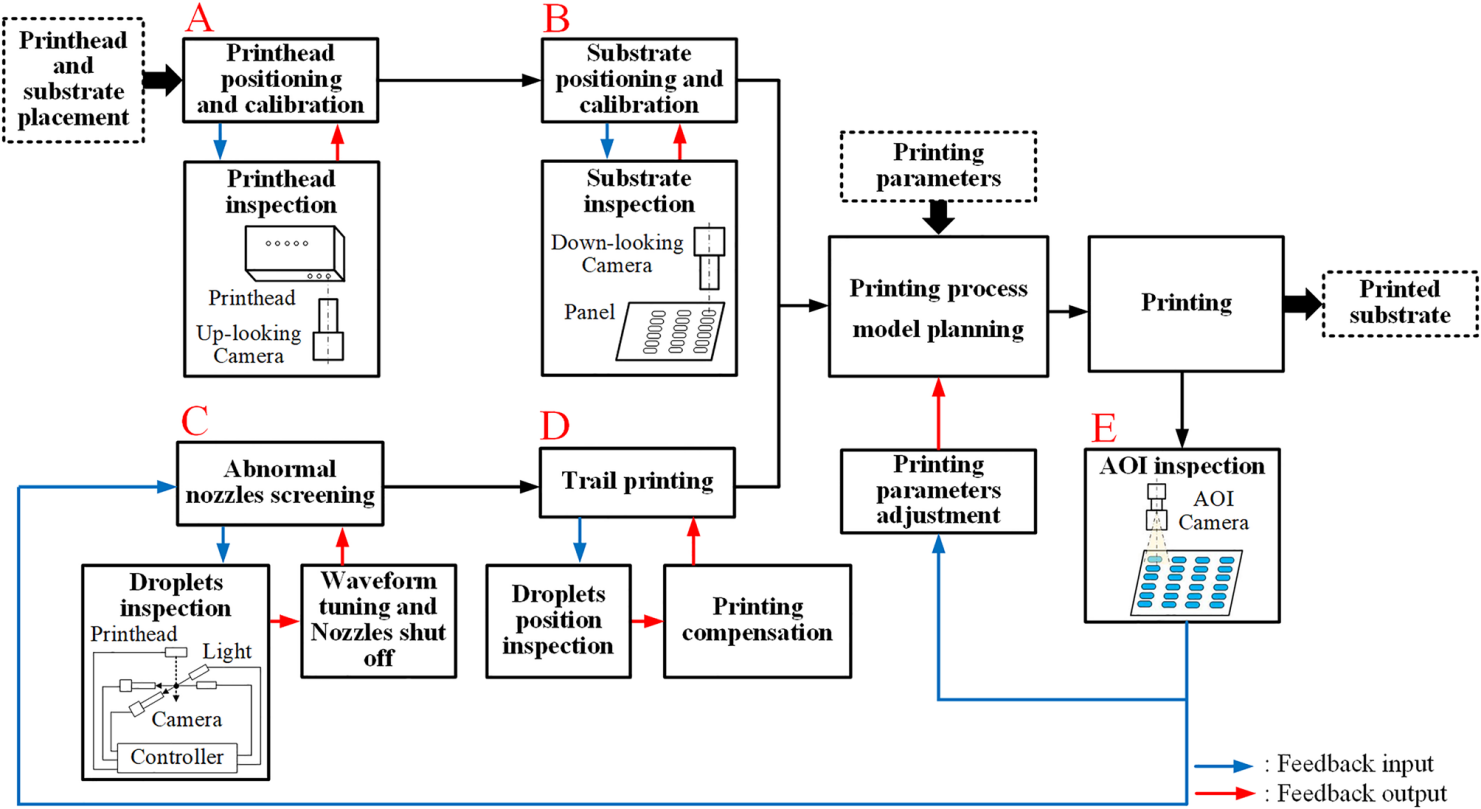
Simplified diagram of the closed-loop feedback system in the printing system.

The closed loops in the printing process flow are designed as shown in Fig. 5, and they include four inner closed loops at different steps in the proposed process and one batch closed loop for the whole printing process. Each inner closed loop can improve the accuracy of the corresponding process step. A batch closed loop for the complete process flow consists of all the inner closed loops and can iteratively optimize the printing results as the printing batch size increases.

The specific actions and functions of the closed-loop printing process are as follows. Inner closed-loop 1: As shown in part A of Fig. 5, after printhead installation is completed, the nozzle position needs to be detected by the up-looking camera. When the printhead angle error exceeds the threshold, the printhead is reinstalled. Inner closed-loop 2: As shown in part B of Fig. 5, since the substrate transfer manipulator generally cannot achieve the precision of substrate placement at the micron level, it is necessary to detect substrate placement with a down-looking camera. When the placement error of the substrate is outside the given range, the system uses the rotating motor of the substrate adsorption platform for calibration. Inner closed-loop 3: As shown in part C of Fig. 5, the droplet measurement module is used to detect the volume, velocity, and angle of all the droplets ejected from the printhead. If the measured droplet parameters exceed the allowable error ranges, the abnormal nozzles are closed, and their positions are recorded. Inner closed-loop 4: As shown in part D of Fig. 5, a trial printing test is performed to determine the droplet deposition position error. The print pattern is a matrix of droplets with specified spacing. After printing, the down-looking camera is used to collect images from multiple locations in the droplet matrix without repetition. Then, the system obtains all the actual droplet position coordinates through image processing. These coordinates are compared with the ideal droplet position coordinates to calculate the droplet deposition accuracy and perform compensation. Batch closed-loop 5: As shown in part E of Fig. 5, after panel printing, the printing results are inspected with AOI cameras. Then, the causes of the error are traced based on the inspected defect types and locations. Based on the specific cause or causes, the printing process parameters, abnormal nozzle positions, printhead, and substrate positions are adjusted and optimized in the next batch. Through multistep closed loops, the printing system can accurately determine the different equipment errors and perform calibration for each step in the process. Moreover, the complete screening of abnormal nozzles on the printhead provides the foundation for the next step in printing planning and enhances the working condition adaptability for abnormal nozzles.

### Set covering printing planning model

To create a printing system with high adaptability, based on obtaining abnormal nozzle positions on the printhead through a multistep closed loop, the remaining normal nozzles need to be used to complete the printing of all pixels on the substrate. Therefore, a printing planning model is established to plan the movement path of the printhead and the nozzle ejection action so that all pixels can be printed completely in the shortest possible time.

The printing process is as follows. After the nozzle moves to the starting point of printing, the substrate starts to move in the X direction. When the printable region of the pixel on the substrate passes under a normal nozzle, droplets are ejected from the nozzle and deposited to form pixels. According to our other research results, the printable region is the largest area where a droplet can finally flow to form a pixel after it is deposited on a substrate^24^. When all the pixels in the same column in the X direction have been printed, one-pass printing is completed. Then, the substrate returns to its original position, and the printhead moves a certain distance in the Y direction so that the normal nozzles are aligned with other unprinted pixels to start the next one-pass printing process. This process is repeated until all the pixels are printed on the substrate.

**Figure 6:**
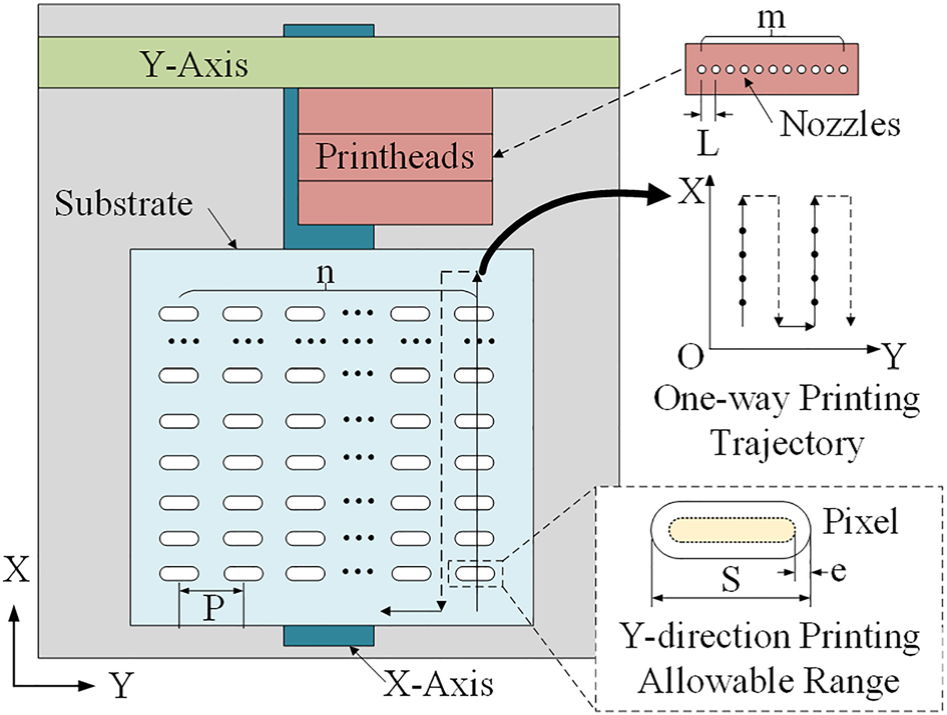
The layout of the display pixel printing system.

Therefore, the printing planning problem is defined as follows. Figure 6 shows the layout and parameters of the display pixel nozzle-array printing system. *L* and *m* are the nozzle pitch and the number of nozzles on the printhead, respectively. *P* and *n* are the pitch and number of pixel columns in the Y direction of the substrate, respectively. *S* is the size of pixels in the Y direction, and *e* is the droplet deposition error in the Y direction. The *S* and *e* together define the Y-direction printing allowable range of the pixel. When the normal nozzle position is within the range, the droplets ejected by this nozzle can be deposited into the pixel. Otherwise, the droplet may deposit outside the pixel and cause printing defects.

According to the pixel printing process, the process is composed of multiple printing passes. The normal nozzles are aligned with different pixels during each pass, and as the substrate moves in the X direction during a pass, all pixels in the column where the aligned pixels are located are printed. Thus, when the position of the printhead for one pass is determined, according to the alignment of the normal nozzle and the positions of unprinted pixels, the indexes of the nozzles that need to be used in this pass can be obtained. Therefore, the printhead position printed per pass is the decision variable of the print planning problem. Moreover, the challenge to be solved is to expect substrate printing in the presence of abnormal nozzles, so it is a strong constraint for the model that the substrate printing can be completed normally regardless of the number of abnormal nozzles. For optimization goals, when the printing speed and the number of pixels in the X direction are determined, the printing time required for one pass is known. The fewer the number of printing passes, the less time it takes to complete the printing job. Therefore, the optimization goal of the printing planning problem is to minimize the number of print passes.

In summary, the printing planning problem can be defined as follows based on the printing process. Consider a printing system that includes one or more printheads with determined relative positions of nozzles and a substrate with a certain pixel pattern. The printhead starts printing the first pass from a certain position on the substrate. Each normal nozzle can be assigned to jet or not when it is within the pixel range, and all abnormal nozzles are not ejected under any circumstances. When there is a nozzle in the pixel range, all pixels in the same column on the substrate are printed along a linear path in the X direction in this pass. Then, the printhead moves a certain distance in the Y direction to print other columns of pixels and performs the next pass. This process is repeated until all pixels are printed. The object of the printing planning problem is to minimize the number of printing passes required to print all pixels.

The problem parameters for the display pixel printing planning problem are shown in Table 1. The problem can be mathematically formulated as the following set covering model:

**Table 1:** Problem parameters for the display pixel printing planning problem.

Variable	Definition	Index	Definition
${{\varvec{a}}}_{{\varvec{i}},{\varvec{j}}}$	0–1 variable; When the first nozzle in the printhead is aligned with the pixel $j$, the element is equal to 1 if there is a normal nozzle in the printing-allowable range of the pixel $i$; otherwise, the element is equal to 0	$i$	Index of pixel columns, *i* ∈ *N*
		$j$	Index of the first nozzle Alignment pixel column, *j* ∈ *N*
		$n$	Number of pixel columns
		$N$	Index set of pixel columns, equal to $\left\{1, 2,\cdots ,n\right\}$

Decision variable	Definition		
${{\varvec{x}}}_{{\varvec{i}}}$	0–1 variable; if the printhead performs one-pass printing when the first nozzle is aligned with the pixel in column $i$, the variable is 1; otherwise, it is 0		

1 $$ Z = min\mathop \sum \limits_{{i = 1}}^{n} x_{i}  $$

2 $$ \mathop \sum \limits_{{i = 1}}^{n} a_{{i,j}} x_{i}  \ge 1,\left( {\forall j \in N} \right) $$

3 $$\begin{array}{c}{x}_{i}\in \left\{\mathrm{0,1}\right\},\left(\forall i\in N\right)\end{array}$$

where Eq. (1) is an objective function to minimize the number of selected stop points, which means that the number of printing passes is minimized. Equation (2) is a constraint to ensure that every pixel column is printed at least once. Equation (3) represents the boundary of the decision variable, which means that each stop point is selected at most once.

The key to the establishment of model constraints is the calculation of ${a}_{i,j}$. The corresponding formula is as follows:

4 $$\begin{array}{c}{a}_{i,j}=\left\{\begin{array}{c}1\qquad \left|{y}_{i}^{px}-{y}_{j,q}^{ph}\right|\le \frac{S-2e}{2}\\ 0 \qquad\left|{y}_{i}^{px}-{y}_{j,q}^{ph}\right|>\frac{S-2e}{2}\end{array}\right.\end{array}$$

where ${y}_{i}^{px}$ is the Y-direction position coordinate of a pixel in the $i$th column and ${y}_{j,q}^{ph}$ is the Y-direction position coordinate of the $q$th normal nozzle on the printhead when the first nozzle on the printhead is aligned with a pixel in the $j$th column. The position coordinates of the abnormal nozzles are not added to ${y}_{j,q}^{ph}$. The starting point of the ${y}_{j,q}^{ph}$ position coordinate is the position at which the first normal nozzle on the printhead is aligned with the pixel in the first column on the substrate. The left-hand side of the condition in Eq. (4) represents the position deviation between the nozzle and the pixel center point. The right-hand side is the error bound of this deviation. When the nozzle position is at stop point $j$, if there is a normal nozzle in the error bound of the pixel in column $i$, the value of ${a}_{i,j}$ is 1; otherwise, it is 0.

The principle of the SCPP model is as follows:

1. According to the number of pixel columns, several Y-direction stop points for the printhead are set in the printed pixel area of the substrate.
2. The alignment of normal nozzles and pixels on each stop point is expressed by ${a}_{i,j}$. After all the values of ${a}_{i,j}$ are obtained, some stopping points are selected by solving the decision variable ${x}_{i}$ of the SCPP model to ensure that there is at least one normal nozzle in the error bound of each pixel column on the substrate in the whole printing process, as shown in Eq. (2).
3. Since Eq. (2) usually has many groups of solutions, Eq. (1) is used to select the solution that minimizes the stopping points in the printing process and take this solution as the result of the model output. This ensures that the printing time is minimal while all the pixels are printed.

Therefore, in a mathematical sense, after the input variables are determined and the SCPP model for this printing is established, if the model has a solution, it means that the normal nozzle on the nozzle can achieve the printing of all pixels on the substrate, and the output objective function $Z$ represents the minimum number of stopping points required to complete the printing of all pixels. The minimum number of stops required is equal to the minimum number of printing passes, which means the minimum printing time. If there is no solution to the model, the normal nozzles on the printhead cannot complete the printing of all pixels on the substrate, and the printing conditions need to be improved to establish a new solvable SCPP model.

According to the objective function $Z$ and decision variable ${x}_{i}$ obtained by solving the model, the minimum number of passes required for this printing and the stop point selected for completing the printing can be obtained. By combining the selected stop point ${x}_{i}$ and the matrix ${a}_{i,j}$, the ejection action of all nozzles at each selected stopping point of the nozzle can be obtained. The printing motion route can be obtained by the position of each stop point selected. According to the printhead ejection action and printing motion route, the nozzle module and motion module of the printing equipment can be guided to complete the printing of all pixels on the substrate.

In addition, since the SCPP model takes normal nozzle coordinates, pixel coordinates, and error bounds of the pixel as input variables, the model can be applied to different printhead types, different abnormal nozzle numbers and positions, and different printed display pixel patterns by changing the values of these variables. Furthermore, the SCPP model is an integer programming model, so the solution obtained by the exact algorithm can be guaranteed to be the global optimal solution^25,26^, which means that the printing scheme with the shortest printing time can be obtained through the SCPP model to complete all the pixel printing.

### Model solution and numerical results

To solve the display pixel printing planning problem, a solution program was developed. The modeling of the display pixel printing planning problem is based on the classic set covering model, which is a classic nondeterministic polynomial hard (NP-hard) problem in integer programming. A variety of algorithms, such as the interior point method^27^, column generation method^28^, heuristic algorithm^29,30^, and some commercial optimizers^31,32^, have been applied to solve this type of problem. Due to the large printing area, many stop positions need to be set for printing planning, which leads to a large problem scale in actual application cases. Therefore, the popular general-purpose 0–1 integer programming optimizer Gurobi is selected to solve the model, and it is especially suitable for solving large-scale linear problems^31^.

To verify the adaptability of the proposed model and solution program in different printing situations, a variety of printing cases are used for testing. The default test parameters listed in Table 2 are obtained from the manufacturing process from 72 pixel per inch (PPI) to 400 PPI display panels, which are widely used in televisions and mobile phone. For tests involving different parameter cases, the adjustment results for all new parameters are not given because most parameters are the same, except those that are discussed. Hereafter, the parameters listed in Table 2 are used in all cases unless otherwise specified. All input parameters come from the actual display panel data.

**Table 2:** Default calculated parameters for printing planning.

Parameter	Substrate PPI	P (µm)	n	S (µm)	e (µm)	Abnormal nozzle proportion
Value	72 PPI	351	570	136	± 10	5%
	85 PPI	300	667	130		
	144 PPI	175.5	1140	125		
	200 PPI	126	1588	114		
	350 PPI	71.4	2802	48.4		
	400 PPI	64.4	3106	35.3		

**Figure 7:**
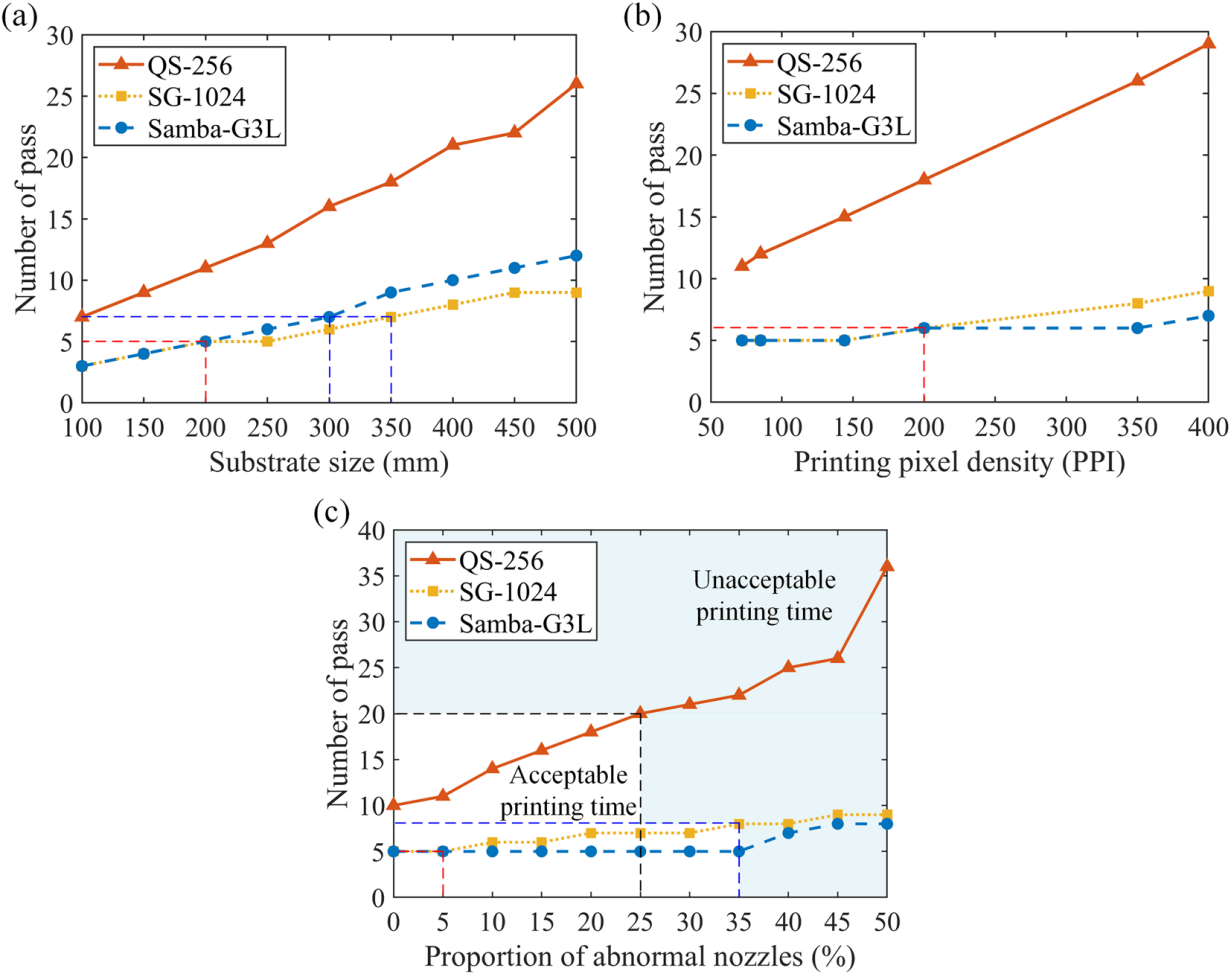
Simulation results of different parameters on the number of printing passes. (**a**) Effect of the substrate size (in the case of 72 PPI substrate). (**b**) Effect of the printing pixel density. (**c**) Effect of the abnormal nozzle proportion on the printhead (in the case of 72 PPI substrate).

The results for different cases are shown in Fig. 7. Since all simulation results are guaranteed to meet the constraint that all pixels on the substrate have completed printing, the objective function $Z$ is chosen as the showing result. To facilitate understanding, the number of passes is used to represent the meaning of $Z$ in the figure. To verify the high adaptability of the model to different printheads, three types of printheads commonly used in industrial printing are tested, including the FUJIFILM Dimatix QS-256 printhead (256 nozzles, 100 DPI), FUJIFILM Dimatix SG-1024 printhead (1024 nozzles, 400 DPI) and FUJIFILM Dimatix Samba-G3L printhead (2048 nozzles, 1200 DPI). By using different types of printheads for printing under different conditions, such as different substrate sizes, pixel densities, and proportions of abnormal nozzles on the printhead, the SCPP model can adapt to different working conditions and complete display pixel printing.

The influence of printing parameters on the SCPP model is numerically explored. As shown in Fig. 7a, usually the larger the substrate size is, the longer the printing time. The number of pass with a low DPI printhead is significantly more than that with a high DPI printhead because only a few nozzles on the printhead can align with the error bound of pixels. For the result of 7 passes, the high DPI printhead. For example, when both print 7 passes, the substrate size printed by the high DPI nozzle is more than 3 times that printed by the low PPI nozzle. However, the DPI of the printhead is not the higher the better. For the high PPI printheads, when the size of the printed substrate exceeds 200 mm, a printhead with a wider print width, such as SG-1024, can complete display pixel printing faster than a printhead with a higher DPI but narrower print width, such as Samba-G3L. As shown in Fig. 7b, different high DPI printheads take similar printing times when printing substrates with PPI less than 200. However, when printing on a higher PPI substrate, the printhead with a higher DPI can complete printing in a shorter time. As shown in Fig. 7c, the results give a parameter space for the proportion of abnormal nozzles in which the different printheads can complete substrates printing in an acceptable time. The printhead with low DPI are more sensitive to changes in the proportion of abnormal nozzles because fewer nozzles of printhead can participate in printing. When the proportion of abnormal nozzles is more than 25%, the QS-256 printhead is recommended to be replaced or maintained because the printing time is twice that of the case without abnormal nozzles. For the high PPI printheads, such as SG-1024 and Samba-G3L, when the proportion is less than 5%, the printing time is not affected. When the proportion is more than 35%, the printing time will increase significantly and the printhead suggests replacement or maintenance.

The numerical results verify the adaptability of the SCPP model to different types of nozzles, different PPI substrates, and different proportions of abnormal nozzles on the printhead. Moreover, it also shows that the SCPP model can be used to simulate the printing results of the printing condition, and the system can choose the appropriate nozzle type and determine the abnormal nozzle screening boundary according to the type of substrate.

All experiments were programmed in MATLAB 2019b and implemented on a personal computer (CPU: Intel Core i7-9750H 2.6 GHz; RAM: 16 GB; OS: Windows 10).

## Results and discussion

### System application

**Figure 8:**
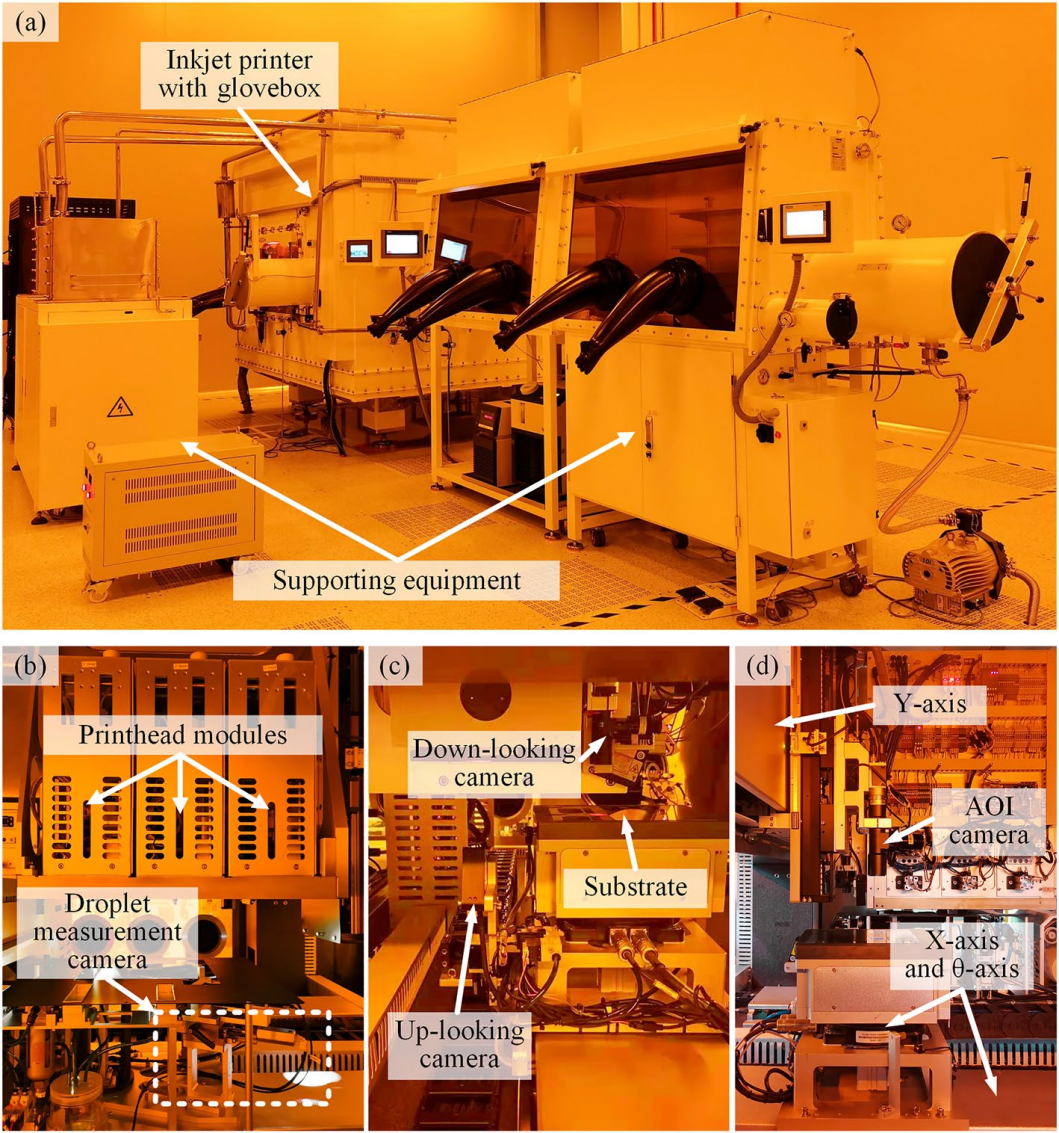
Inkjet printing system for printed display mass production. (**a**) The system with a glove box and supporting equipment. (**b**) Printhead module and droplet measurement camera. (**c**) Down-looking camera and up-looking camera. (**d**) AOI camera and motion module.

To evaluate the practical printing effect and application performance of the proposed method, self-developed printing equipment using the display pixel nozzle-array inkjet printing system and SCPP model is designed and built. As shown in Fig. 8, the printing equipment consists of three printhead modules, a multiaxis motion module, multiple visual inspection modules, glove boxes, and other accessories that assist in printing. As shown in Fig. 8a, the inkjet printer is the main device used to complete the printing process, and it is equipped with supporting components such as glove boxes and other equipment used to complete the subsequent processes. As shown in Fig. 8b, according to the relevant droplet volume and ejection frequency requirements in the display pixel printing process, a FUJIFILM Dimatix QS-256 printhead, which is composed of 256 nozzles with 100 DPI and a drop volume of 4–12 pl, was selected. The droplet measurement module included two droplet-watching cameras, and high measurement accuracy was achieved; notably, the droplet measurement system has a maximum volume measurement deviation of 3%, which is more accurate than other droplet measurement systems of the same type. As shown in Fig. 8c, the down-looking camera was installed next to the printhead module, and the up-looking camera was installed near the X-axis. As shown in Fig. 8d, the X-axis and Y-axis motion systems used linear motors to realize the horizontal linear motion of the different modules and substrate, and the θ-axis was used to rotate the substrate carrying platform to correct deviations in the substrate placement angle. The AOI cameras could independently move along the Y-axis for scanning and inspection of the printing results. The display pixel printing system can achieve a printing speed range of 0–450 mm/s, a maximum substrate size of 200 × 200 mm, and a maximum droplet ejection frequency of 50 kHz; therefore, it can adapt to different pixel pattern requirements and industrial-grade printing manufacturing conditions.

To ensure the reliability of the printing process, the printing equipment improves the stability of the printing system through the design of the ink supply system, the printhead waveform optimization, and the maintenance operation in the printing gap. These designs and operations enable the equipment to maintain a normal working state for a long time. When the AOI camera does not find defects in the printing result, the equipment can periodically perform printhead detection and printing planning while printing the same substrate. When the AOI camera detects defects in the printing result, the printing system can trace the specific abnormal nozzle position through the mapping produced by the printing planning according to the position of the defects. Based on the current proportion of normal nozzles, the system can automatically choose to suspend printing to close the abnormal nozzles and rerun the printing planning, or stop printing for abnormal nozzle screening.

**Figure 9:**
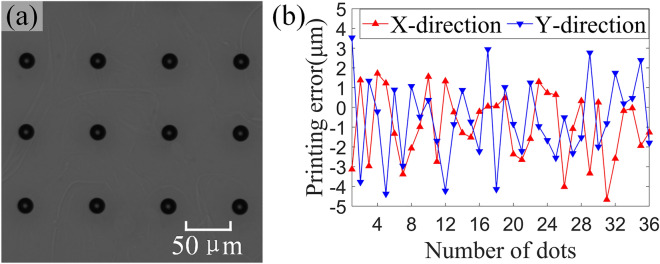
Droplet deposition accuracy. (**a**) Droplet deposition positions. (**b**) Droplet deposition accuracy in the X direction and Y direction.

The droplet deposition accuracy is an important index of the display pixel printing system. Because pixel size is determined by substrate, Eq. (4) shows that when droplet deposition accuracy is smaller, the error bound of the pixel can be larger, and more nozzles can align with pixels and participate in printing. Therefore, an experiment was designed to test the droplet deposition accuracy of the proposed display pixel printing system. The ink was printed on a nonpixel indium tin oxide (ITO) glass substrate that had undergone hydrophobic treatment. The printing pattern was a rectangular array of 6 × 6 ink droplets, the pitch between dots was 100 μm, and the printing speed was 100 mm/s. According to the proposed printing system and planning model, the droplet deposition accuracy experiment was performed after multistep visual inspection and abnormal nozzle screening. As shown in Fig. 9a, the position of the droplets was inspected with a down-looking camera, and the coordinates of the droplets were compared with the theoretical positions. The statistical results are shown in Fig. 9b. The X-direction droplet deposition accuracy is ≤  ± 4.9 μm, and the Y-direction droplet deposition accuracy is ≤  ± 4.4 μm. These droplet deposition accuracies can meet the printing requirements of the display below 200 PPI; thus, the system can meet the pixel density requirements of most TVs and display screens in the market. When the pixel density of a panel is higher than 200 PPI, such as for the panels of mobile phones, the pixel size may be smaller than 30 μm. In this case, a droplet deposition accuracy of ± 5 μm would not be sufficient for ensuring the stability of the printing process.

### Device printing experiment

**Figure 10:**
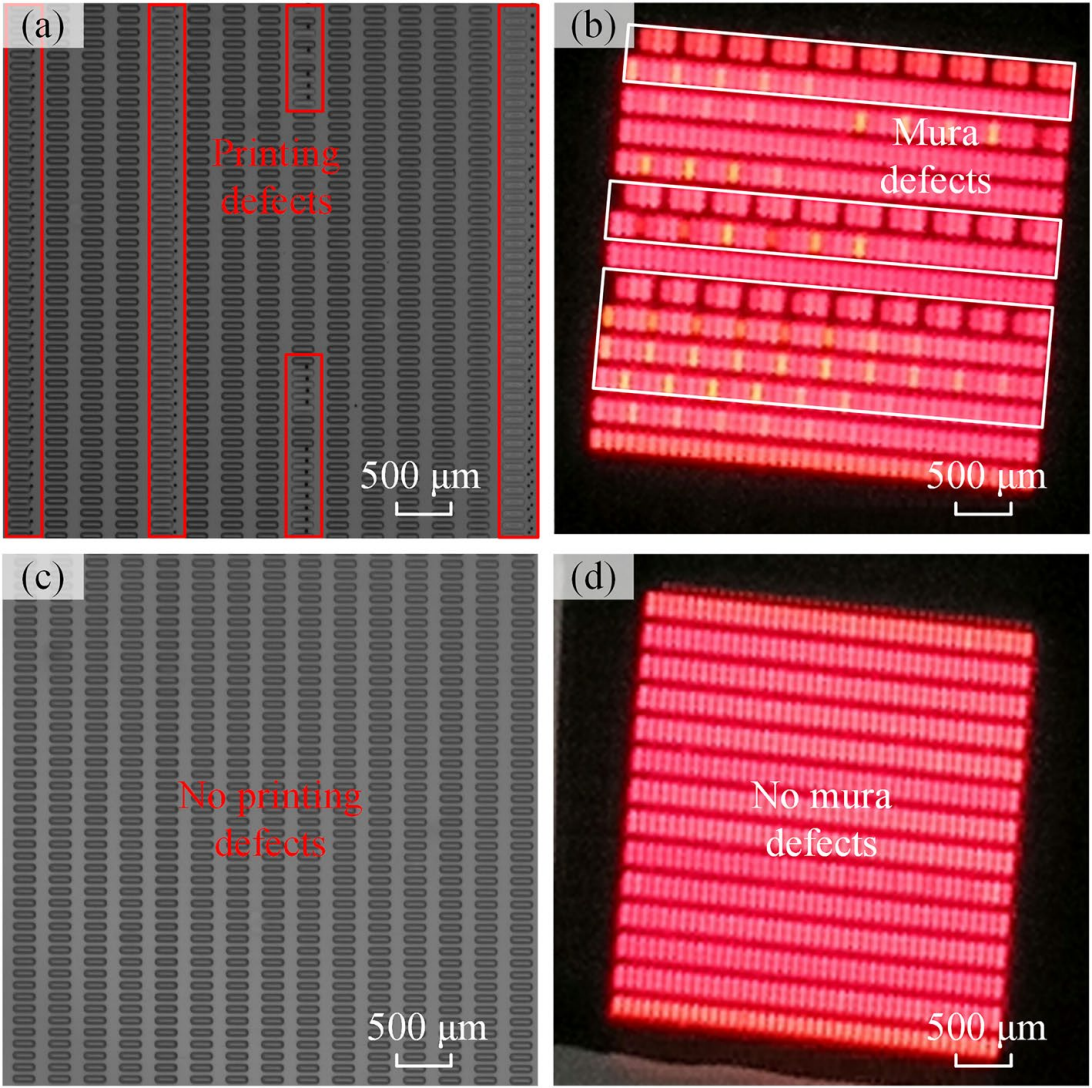
Printing and lighting results for panels with the traditional printing method and SCPP method. (**a**) The defective printing result from the traditional printing method. (**b**) Defective lighting resulting from the traditional printing method. (**c**) The defect-free printing result from the SCPP method. (**d**) The defect-free lighting result from the SCPP method.

To verify the performance of the proposed inkjet printing system and SCPP model, a comparative device printing experiment with and without the SCPP model was tested. The printing parameters of the comparative printing experiments are shown in Table 3. Through multistep visual inspection, 12 abnormal nozzles were found on the printhead. The results are shown in Fig. 10. Figure 10a and b show the printing and lighting results of the traditional printing method. Numerous printing defects can be found in Fig. 10a, such as missing printed pixels and droplets deposited outside the pixels on the printed substrate. These defects cause mura defects after the panel is lit, as shown in Fig. 10b. The reason for the defects is that there are abnormal nozzles involved in the printing process. For instance, the inclined trajectory of the droplets ejected from the nozzles causes the droplets to be deposited in the wrong positions so that the droplets cannot be deposited into the pixels. As a comparison, Fig. 10c and d show the printing and lighting results with SCPP under the same printing conditions. Through the shielding of abnormal nozzles and printing model planning, no printing defects or mura defects are found in the results. The above comparative experiments demonstrate the effectiveness of the proposed printing planning model and system in reducing printing defects.

**Table 3:** Printing parameters for comparative printing experiments.

Parameters	Value
Pixel density	84
Pixel size	180 × 60 μm
Pixel pitch	300 × 100 μm
Printing size	200 × 200 mm
Printing speed	100 mm/s
Abnormal nozzles	5, 27, 30, 41, 58, 94, 125, 182, 189, 224, 233, 251

**Figure 11:**
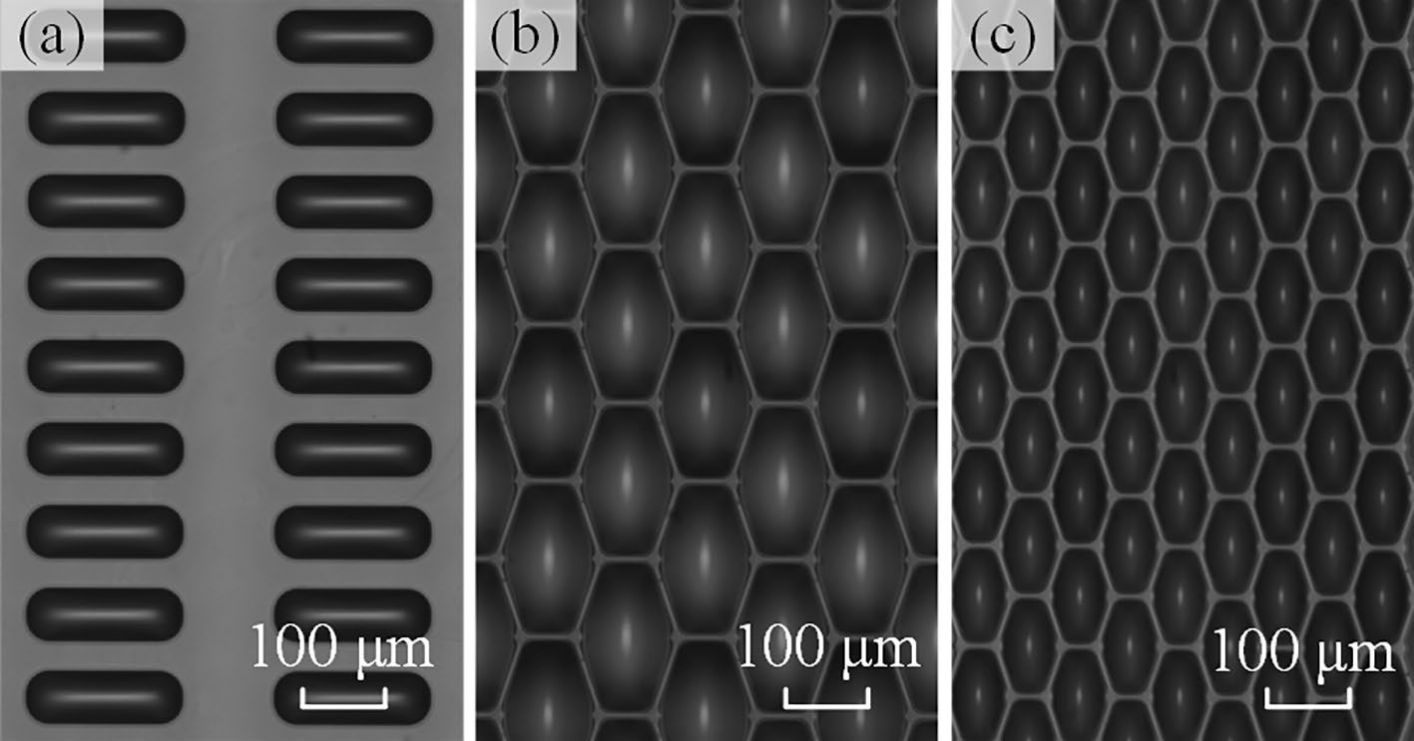
Printing results for panels with different pixel densities. (**a**) 85 PPI. (**b**) 144 PPI. (**c**) 200 PPI.

To verify the adaptability of the proposed system for printing different PPI substrates, experiments were carried out on ITO substrates with different pixel patterns using the proposed jet printing system with the SCPP model. The parameters of the substrate and printing process are shown in Table 4. The printing results are shown in Fig. 11. Figure 11a–c show the 80 PPI, 135 PPI, and 200 PPI panel printing results recorded with the down-looking camera, respectively. The results indicate that the HIL layer of the 400 cm^2^ OLED device printed with the proposed printing system and planning model exhibits no printing defects. The numbers of printing passes are 50, 80, and 90, and the maximum printing time does not exceed 240 s, which meets the time requirements of the display printing processes. These findings verify the high adaptability of the proposed method to different types of pixel substrates.

**Table 4:** Printing parameters for panels with different pixel densities.

Parameters	Case 1	Case 2	Case 3
Pixel density	84	135	200
Pixel size	180 × 60 μm	96 × 96 μm	56 × 56 μm
Pixel pitch	300 × 100 μm	188 × 188 μm	126 × 126 μm
Printing size	200 × 200 mm		
Printing speed	100 mm/s		
Abnormal nozzles	36, 39, 51, 58, 63, 66, 88, 90, 128, 130, 141, 150, 179, 193, 209, 216, 229, 238, 246		
Printing passes	50	80	90
Printing time	122 s	195 s	219 s

**Figure 12:**
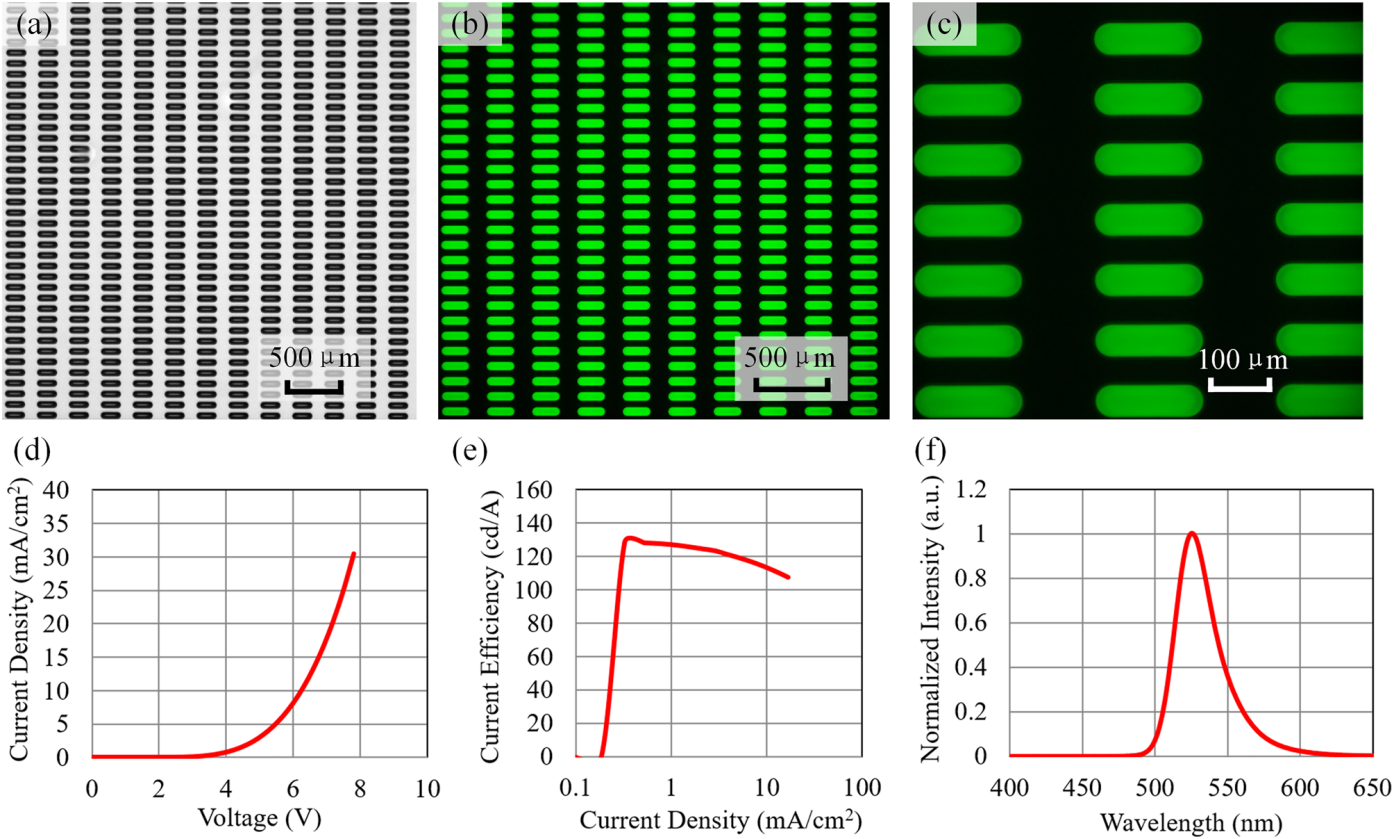
Printing and lighting results for a 400 cm^2^, 85 PPI OLED panel. (**a**) Printing results. (**b**) Lighting results. (**c**) Illuminated pixels under a 5 × microscope. (**d**) J–V curve of the printed device. (**e**) CE–J curve of the printed device. (**f**) EL spectrum of the printed device.

Figure 12 shows the lighting effect of the printed device using the proposed system and model. The printed device exhibits no defects, and the pixels are evenly filled with ink, as shown in Fig. 12a. The lighting result is shown in Fig. 12b and c. The device emitted light uniformly without prominent pixel damage. Figure 12d–f shows the characterizations of the printed device, including the current density–voltage (J–V) curve, current efficiency—current density (CE–J) curve, and electroluminescence spectrum (EL-spectrum). The current efficiency of the device can achieve 127 cd/A under a voltage of 4.2 V. The wavelength peak is 526 nm. The characterizations of the printed device met the quality requirements of the display. This result further verified the application effect of the proposed printing system and printing planning model.

## Conclusion

In this paper, a highly adaptable display pixel nozzle-array inkjet printing system based on an SCPP model is proposed. This system and planning model can overcome abnormal nozzle ejection issues and can improve the adaptability and scalability of printing technology applied in printed display industrial production. First, a display pixel nozzle-array inkjet printing system based on multistep visual inspection and a closed-loop feedback process is designed to monitor the droplet state and provide feedback during the whole process from preprinting to postprinting and accurately identify the positions of abnormal nozzles. Second, an SCPP model is proposed, which can be applied in printing planning for any abnormal nozzle position, any printing pattern, and any type of nozzle condition. The model uses the results of abnormal nozzle screening and the printing pattern as inputs and outputs of the multipass printing path and nozzle ejection actions. Based on these planning results, the system can realize high-precision, high-adaptability, and high-efficiency display pixel printing by using a nozzle-array printhead. Additionally, display pixel printing equipment is established according to the proposed system and model, and a series of experiments are conducted on the equipment. The experimental results show that the proposed display pixel printing system and printing planning model can achieve a droplet deposition accuracy of ≤  ± 5 μm. With this equipment, the printing of substrates with various pixel densities and the printing and lighting of a 400 cm^2^, 85 PPI OLED device are achieved. The printing and lighting results are satisfactory, and the system can meet the current application requirements of the display printing and manufacturing industry. The technology is highly adaptable and scalable and can be used in the industrial-grade printing manufacturing of large-area printed display panels by increasing the number of printheads. This technology can also be applied in other sectors of printed electronics manufacturing.

## Materials and methods

### Materials

The OLED HIL, HTL, and EML ink material was purchased from the DuPont company.

### Methods

#### Printer setup

The NEJ-PR200 printing equipment was self-developed and built by Wuhan National Innovation Technology Optoelectronics Equipment Company. A QS256 printhead from FUJIFILM Dimatix company is chosen for printing. The printing equipment can realize high-precision visual positioning, accurate stereo-vision-based flying droplet measurement, adaptive control of process parameters, and online AOI defect automatic monitoring. Through these functions, the construction of the proposed printing system and the operation of the SCPP model were realized.

#### Characterization

The luminance and spectrum data of the OLED printed devices were measured by using a five-axis optical measurement instrument (CS2000, Konica Minolta).

## Data Availability

All data generated or analysed during this study are included in this published article.
